# Testing Model of Purchase Intention for Fast Food in Mexico: How do Consumers React to Food Values, Positive Anticipated Emotions, Attitude toward the Brand, and Attitude toward Eating Hamburgers?

**DOI:** 10.3390/foods8090369

**Published:** 2019-08-27

**Authors:** Héctor Hugo Pérez-Villarreal, María Pilar Martínez-Ruiz, Alicia Izquierdo-Yusta

**Affiliations:** 1 Faculty of Economics and Business Studies, University of Castilla-La Mancha, 02071 Albacete, Spain; 2 Engineering and Business Postgraduate Center, Popular Autonomous University of Puebla State, 72410 Puebla, Mexico; 3 Faculty of Economics and Business Studies, University of Burgos, 09001 Burgos, Spain

**Keywords:** food values, positive anticipated emotions, attitude toward the brand, attitude toward eating a hamburger, purchase intention

## Abstract

This research investigated the effect of the food values, positive anticipated emotions, attitude toward the brand, and attitude toward eating a hamburger on purchase intention in fast-food restaurants in Mexico conjointly. The purpose of this study was to discover which variables influenced the consumer´s intention to buy. Data was collected from a survey of 512 Mexicans fast-food consumers. Structural equation modeling was used to test the hypothesized associations. The results showed that food values and positive anticipated emotions absolutely impact the attitude toward the brand, which impacts the purchase intention of the Mexican consumers. Nonetheless, the positive anticipated emotions impact stronger than food values, and the best way to get a purchase intention is toward the attitude of the brand rather than attitude toward eating a hamburger. The authors discussed inferences and suggestions for consumer approaches.

## 1. Introduction

Food choice decisions are complicated when every day the consumers make a lot of decisions about one excellent fast food [1]. Over the past few years, some studies have had a primordial objective to explain how interaction facts affect purchase intention through theory planned behavior (TPB) [2,3,4]. However, none focused on the food values, especially when the research was about food choice and positive anticipated emotions like a central variable in the model. Based on a dataset of 1169 abstracts of marketing from 2005 to 2014, Barahona et al. (2018) [5] explained that one crucial dimension for researchers is emotional marketing. Topics such as evaluation, experience, message, people, emotional, goal, and hedonic are the keywords for studies in this field. Therefore, this research was based on the purpose of explaining the purchase intention in four main premises. First, fast food consumption has a purchase intention by the attitude toward the brand into the means of an emotional need according to a physiological desire [6,7,8]. Second, the consumers´ emotions influence the purchase intention [9]. Third, what is the role of food values on attitude toward the brand and attitude toward eating a hamburger [10]? Fourth, what is more essential to predict the purchase intention: attitude toward the brand or attitude toward eating a hamburger [11]?

Through this research, a model with these variables was proposed because there is a synergistic effect between them. The approach rests with the effects of food values and positive early emotions directed towards the form of the attitude as a predecessor of the purchase intention [12,13,14]. This model was designed from the separation of attitudes: one directed towards the act of eating and another towards the brand. The application covers the principle on attitudes directed towards the product and another towards the brand. Thus, this model is the first that uses the rational and emotional part of consumption and separates the attitude of eating from the attitude towards the brand. In this case, the model provides information on the importance of the product and the brand and towards launch, modifications and valuations of products and brands. The consumer’s decisions are based on some level of rational or emotional effect [15,16].

This study forms the rational (food values) and emotional (positive anticipated emotions) parts to connect them with different attitudes to predict purchase intention. Consequently, it used these two attitudes roles, eating versus brand, to test the relationship to purchase intention. The importance of the study is to predict the purchase intention and to know the consumers’ behavior choices with a hamburger. If the calculations, weights, loadings, etc. contribute to explaining more of the purchase intention, it should make an important and significant contribution to academic literature. This is because it gives off too many forms to investigates and implement strategies in fast-food restaurants, knowing the protrusion factors in the model.

For these reasons, it is intended to identify which emotions, food values and types of attitudes impact significantly and positively on the purchase intention. Through these findings, marketing strategies can be formulated and it is possible to know what the most convenient way for this field is. The objective of the present study was to explicitly test the purchase intention toward attitudes, food values and positive anticipated emotions. The study built a model on purchase intention research by examining the consumer before the purchase decision. Also, this study emphasized the meaning of the role of attitudes (eating hamburger and brand) on purchase intentions of fast food consumers. Finally, the study tested and confirmed the hypotheses planted in this research.

### 1.1. Attitudes in Consumer Behavior 

Attitude toward something is an antecedent of intention, but it is also the degree to which an individual has a favorable or unfavorable evaluation or appraisal of the behavior to any purchase situation [17]. Some research has also highlighted the role of purchase intention and the attitude impact [18]. On the other hand, the attitude that is formed in the first stage is formed of the decision process of purchase in the consumer (recognition of the need/problem). Some studies proved that the attitude directly affects the consumer’s buying behavior [19,20,21]. This attitude is influenced by elements such as information, nature of the product, social media, ads and other behavioral factors. In the context of food consumption, the role of attitudes is at the top for research in consumer behavior. Thus, some consumers have attitudes toward eating hamburgers and others have attitudes toward the brand. This is because they keep both positive and negative evaluations, such as purchases intentions, purchases and repurchases [22]. However, in marketing as a discipline, the gap is different between attitude toward eating a hamburger and attitude toward the brand.

Attitudes toward eating hamburgers play a significant role in understanding consumer behavior. These attitudes can be decision-making components for the choice and intention to eat some food [23,24]. Once consumers recognize their need for food, they enter into a stage of searching and evaluating the alternatives [25]. It is at this stage, where people positively or negatively value the desired behavior without implying the degree of eating habits or the level of hunger [26]. Hence, the attitude of eating evaluates the favorable or unfavorable predisposition towards the act of eating any food [17]. Rezai et al. (2017) [27] pointed to a direct relationship between attitudes towards eating foods that generate a healthy benefit and the intention to buy. For this reason, it is vital to know one’s attitude towards the act of eating as a central point towards the intention to buy.

On the other side, attitudes are cognitions and can sometimes be directed towards the brand [28]. So it is necessary to comment that attitudes towards the brand can generate a behavioral intent and the same behavior of the consumer’s final purchase [29]. Therefore, attitudes towards the brand mean that consumers adopt or reject conduct based on experiences, personal recommendations and media exposure, as well as other media that use the brand and may have a point of contact with the consumer [30]. Hence, attitudes towards the brand have become one of the intangible components valued by consumers because when choosing the behavior, they do it more for the brand than for the product. Similarly, the attitude towards the brand makes consumers acquire feelings of security, confidence, convenience, and credibility among others, so for them, it is easier to recognize and choose the purchase [31]. Thus, the literature agrees that attitude towards the brand is the highest point through which the consumer disseminates the choice.

### 1.2. Purchase Intention

Assael (1998) [32] called purchase intention the conduct that seeks in response to an object and is before the purchase. Subsequently, Zhang et al. (2018) [33] approved the relationship between attitudes and purchase intention. Phau and Teah (2009) [34] demonstrated that when the consumer has a strong positive attitude, there is a higer intention to buy.

Rezai et al. (2017) [27] pointed out the importance of determining the intention to purchase functional products from examining the factors involved in the purchase decision process. For example, Jahn, Tsalis, and L’hteenm-ki (2019) [35] indicated that the general attitude towards products has a direct effect towards the intention to purchase, as long as the people are in a condition of suitability and knowledge of the problem. Asif et al. (2018) [36] pointed out that it is possible to find differences in intent to buy from one country to another, but they agreed that attitude and health awareness are the best predictors of the intention to buy in organic foods. Some studies pointed to some additional variables to the TPB including moral attitude and healthy awareness towards purchasing intent in organic foods [37]. Consequently, it is possible to include other variables in the purchase intention by extending the TPB. On the other hand, another study pointed to the involvement towards the consumption of products, price sensitivity and moderation of the effect of the identity of the local product towards the intention of purchase [38].

Chiu, Hsieh, and Kuo (2012) [39] and Diallo (2012) [40] underlined aspects about the probability to buy, not before the consumer formed an attitude and experience of the past. Now, as the intention is testified to be a significant factor of buying, it was thus, hypothesized that:

**Hypothesis** **1** **(H1).** *Attitude toward the brand will positively influence intention to buy.*

**Hypothesis** **2** **(H2).** *Attitude toward eating hamburger will positively influence the intention to buy.*

### 1.3. Food Values

The situation of obtaining information on the attributes of the product has always been a relevant topic in food consumer research. Today, exotic consumption attributes, towards the ethics of consumption, healthy awareness, animal impact and organic food are topics of interest in knowing one’s behavior [41,42,43,44]. According to Basha and Lal (2019) [45], the ratio of environmental concern, health and lifestyle, supporting local farmers, product quality, convenience, price, animal welfare, safety-trust, subjective norms, and attitude is valued. The food choice has been becoming an advantage to improve healthy and sustainable diets and to know the different roles of high and low involvement [46]. Nevertheless, Boer and Schösler (2016) [46] mentioned that the differences in the affinities could be predicted by food-related value motivation.

Sprotles and Kendall (1986) [47], through consumer styles inventory (CSI), claimed that consumers choose to make their purchase decision through eight basic styles: high quality, innovation, brand awareness, price, hedonism, confusion with other brands, impulsivity, and habit. Other studies emphasized product presentation, food safety, environmental impact, and ethical consumer identity [48]. Another study found that depending on the type of food (organic or conventional) used, the effect on the consumer perception component (e.g., healthy consciousness) differs [49].

When researches talk about the food attributes, it can be partial to the real concept because the food attributes can be an infinite number of characteristics, but only some of them are important for the moment of choice [50]. For this reason, the attributes of the product became the consumer’s values regarding food. Some researchers affirmed that these values were influenced through many factors, which relate to personal values [1,51,52,53]. This means that food values are exercised by the consumer and not by the product itself. However, each attribute mentioned above falls within a factor of the 11 described by Lusk (2011) [54]. Thus, it is possible that each product, depending on belonging in the category, constitutes intra-group differences, but it is possible to categorize them in general forms.

Lusk and Briggeman (2009) [55] explored all the factors that integrated the attributes of food. After this plan, Lusk (2011) [54] opened wide 11 items to identify the food values scale. These items are (1) naturalness (the extent to which food is produced without modern technologies), (2) taste (the extent to which consumption of food is appealing to the senses), (3) price (the amount paid for food), (4) safety (the extent to which consumption of food will not cause illness), (5) convenience (the ease with which food is cooked and consumed), (6) nutrition (the amount and type of fat, protein, vitamins, etc.), (7) tradition (preserving traditional consumption patterns), (8) origin (where the agricultural commodities were grown), (9) fairness (the extent to which all parties involved in food production equally benefit), (10) appearance (the extent to which food looks appealing), and (11) environmental impact (the effect of food production on the environment). 

Studies have shown that food values are essential to explain attitudes. For example, Manan (2016) [1] emphasized to know the attitudes through personal values, but the question is whether personal values are influenced by the food benefits, if that correct, then these affect attitude. In order, Lang and Lemmerer (2019) [53] demonstrated the relationships across personal values and attitudes toward local food, but they did not separate the attitude toward eating a hamburger or the attitude toward the brand. As a result, it is hypothesized that:

**Hypothesis** **3** **(H3).** *Food values will positively influence attitude toward the brand.*

**Hypothesis** **4** **(H4).** *Food values will positively influence attitude toward eating a hamburger.*

### 1.4. Anticipated Emotions

Some researchers have been in charge of framing emotions as a fundamental, principal axis and detonator of all purchasing behavior, this adding to the part of information processing and consumer action [56,57,58,59,60,61,62]. Although the entire chain of observation (cognitive, conative and affective), the trigger and the key factors of success cannot be established, some researchers have taken a part of the chain towards the effective and successful verification of the application of branding emotional, buyback, purchase decision, search, and evaluation of purchase alternatives [63,64,65,66].

Within the contributions of advertising, it is possible to highlight that the emotional contagion may have main effects on the physiological changes of the people [67]. In this study, the participants felt sadder when they saw a victim with a sad face, and their sadness emanated the effect on the expression of the emotion in the sympathy. The effects of contagion are automatic and not inferential but are diminished by deliberative thinking. On the other hand, Nielsen et al. (2010) [68] showed that the “pre-attention” processing of semantic information in non-focal announcement titles can provoke orientations towards attention responses. The same results were in foreseeable increases in the ad and knowledge of the brand. Equally, Teixeira et al. (2012) [59] showed that surprise and joy concentrate effective attention and retain the viewers with more time. However, the most important thing is the level of retention instead of the speed of surprise, and it affects the concentration of attention more. Therefore, speed influences the level of joy, which affects spectator retention. These three studies placed the emotional part as the main factor in their research with the impact on advertising. It could be specified that the authors discussed the implications of the use of emotional expressions, titles of advertisements, and consumer knowledge of the brand to promote emotions in the consumer and help the purchasing decision process. 

However, the emotions are present throughout the process of consumer behavior, but it is vital to determine what the origin of this is. Pelsmaeker et al. (2017) [69] explained the relationship of emotions in the begging of the process of consumer intention, and they determined the relevance of applying an evaluation before recognizing the need. Emotions can indeed be positive and negative depending on the moment or value. However, some researchers in recent years were working only for positive emotions because only these matter. Wen, Hu and Kim (2018) [70] examined the effect of individual culture on positive emotions for the recommendation intention. Finally, positive emotions are the principal element to determine the satisfaction of the consumer [71]. 

Williams and Aaker (2002) [72] believed that when individuals are exposed to mixed emotions, they influenced the individual´s attitudes in general. They also demonstrated that the detonation of emotions with duality (e.g., sadness and happiness) is less prone to form an attitude towards their behavior. Haws and Winterich (2013) [73] described the factors to measure the attitude toward eating directly to these items: pleasure, enjoy, satisfied, and good taste. However, the consumer can have an attitude toward the brand and not for eating. That reason describes Aggarwal and Mcgill’s (2012) [74] finding that what consumers like, think, admire, and fit in their life is a good positive attitude that helps to stimulate the intention. This study proposed two constructs, one for eating the hamburger and the other for the brand.

Thus, the following hypothesis can be derived:

**Hypothesis** **5** **(H5).** *Positive anticipated emotions will positively influence attitude toward the brand.*

**Hypothesis** **6** **(H6).** *Positive anticipated emotions will positively influence attitude toward eating a hamburger.*

**Hypothesis** **7** **(H7).** *Positive anticipated emotions will positively influence the intention to buy.*

**Figure 1:**
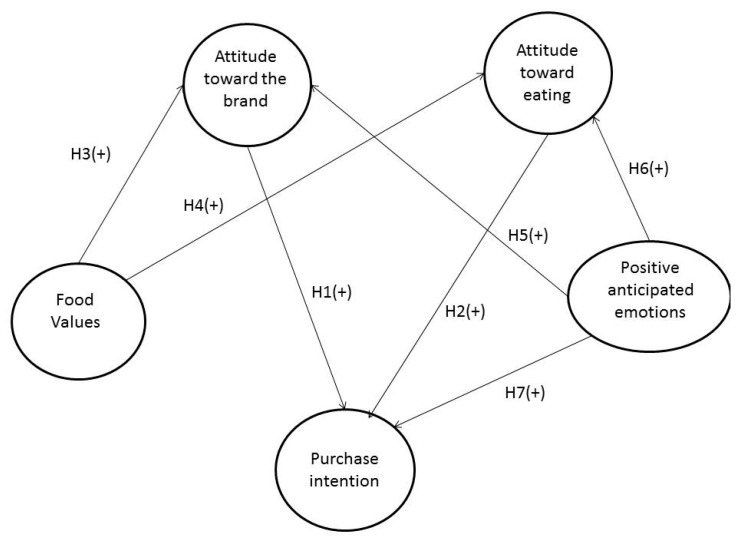
Model development.

Therefore, seven hypotheses were tested in this research and based on the discussion above (see Figure 1), and considers seven proposed effects: (1) attitude toward the brand on purchase intention, (2) attitude toward eating hamburger on purchase intention, (3) food values on attitude toward the brand, (4) food values on attitude toward eating hamburger, (5) positive anticipated emotions on attitude toward the brand, (6) positive anticipated emotions on attitude toward eating hamburger, and (7) positive anticipated emotions on purchase intention. Thus, all the effects correspond to a new model for understanding better the purchase intention in fast-food restaurants. 

## 2. Materials and Methods 

This study utilized partial least squares-structural equation modelling (PLS-SEM) to examine the impact of the food values, emotions anticipated and attitudes on purchase intention (see Table 1 for technical details). The proposal was to estimate a model that includes a mix of factors and composites using the PLS algorithm procedure [75]. The idea was to maximize the explained variance of all dependent variables used in the research model. In this case, the research intent was to know the predictor variable and to identify possible drivers [76,77]. Therefore, the independent variables that the literature reports as important predecessors of purchase intention were also included.

### 2.1. Data Collection 

The data was collected from Puebla City in Mexico with a consumer survey of 512 participants. Participation was voluntary and all of them completed the questionnaire.

### 2.2. Statistics Analysis 

The study used structural equation modeling (SEM) to test the conceptual model with SmartPLS 3.0 software. According to Streukekens and Leroi-Werelds (2016) [78], this study used partial least squares (PLS) with a 10,000 subsample bootstrapping procedure and the same software to know if the relationship was supported or not with the results. In the beginning, this model was composted from 34 items reduced to 28 items in five constructs. From there, no preliminary empirical parameters for this particular market were found. 

### 2.3. Questionnaire Development

The questionnaire was constructed and divided into five sections: (a) food values, (b) positive and negative anticipated emotions, (c) attitude toward the brand, (d) attitude toward eating a hamburger, and (e) purchase intention (see Table 2). The first table shows the questionnaire section by source and the second explains details on how to measure each variable.

The food values utilized a Likert scale 1–5 (1 = not at all important, to 5 = extremely important). The scale was adapted from 7 points to 5 points, because it was planned to explain each item as a formative construct. It is better to get an answer from the consumer on the assumption that some items do not have a relation with the construct. Positive and negative anticipated emotions applied a Likert scale 1–7 (1 = none, to 7 = severe). From the original items, it supported the positive emotions because the negatives did not have an impact and did not comply with the test of validity and reliability. It deleted the emotions for: glad, relief and happy for the reason to have multicollinearity and the VIF factor > 3.2. Also, it used the 7-point Likert scale as the author marked it. According to Becker and Ismail (2016) [80], it is possible to use different Likert scales within the same model. In the attitude toward the brand (ATB), it used a Likert scale 1–5, (1 = strongly disagree, to 5 = strongly agree). From the original contribution, it supported only the positive items because the weights were weak (item 4 “shame” and 5 “avoidance”). It changed the inverse items for the nature of the scale. For the attitude toward eating a hamburger (ATEH), it was handled with a Likert scale 1–5, (1 = strongly disagree, to 5 = strongly agree). These items were adapted to the specific product (in this case, hamburger). The variable purchase intention was measured by a Likert scale 1–5, (1 = strongly disagree, to 5 = strongly agree). PI4 was excluded because it had multicollinearity with PI3. The item was “I would buy in fast food restaurants next time”.

All the constructs were reflective, not including food values. The construct formed the interpretations depending on the dependent variable. Hence, the formative indicators may show up as non-significant. Also, the indicators were correlated with other indicators in the model proposal [81]. Similarly, all the formative indicators required a census of all items for the construct because each one (it can be negative or positive) was formed into a complete variable. Even the negative influences on the consumer were one item that needed to be taken care of [82]. Finally, the overall fit of this model does not matter; the other covariances like the exogenous variables are outside the model proposal, and all the items are independent of themselves, according to Jarvis, MacKenzie and Podsakoff (2003) [82].

## 3. Results

The development model was constructed on an amalgamation of items, concepts, models, effects and principles about two parts: functional and emotional. This model was also composited about a series of research studies around four exceptional areas: (1) food values, (2) attitude toward the brand, (3) attitude toward eating a hamburger, and (4) positive anticipated emotions. All were within the proposal to better explain the purchase intention in fast-food restaurants in Mexico.

To assess the goodness of model fit, the root mean square residual (SRMR) was utilized. According to Hu and Bentler (1998) [83] and Hu and Bentler (1999) [84], SRMR < 0.08 is a good fit for SRMR. This model has an SRMR = 0.049 < 0.08 SRMR criteria; these measures found that this model has a good fit with the parameters mentioned before. The normed fit index (NIF) results in values from 0 to 1, and the closer to 1, the better the fit [85]. In this model, the NIF was 0.899 and represented an acceptable fit.

To get confidence in this model, reliability and construct validity testing were carried out. Cronbach’s alpha coefficient was accepted for all the constructs, having a value greater than 0.7 [86]. The rho_A value was reflected regularly if this index was larger than 0.7 [87]. The composite reliability (CR) values under 0.6 indicated a deficiency of internal consistency reliability [88]. The AVE of each construct was above the tolerability value 0.5 [89,90] (see Table 3).

As a final point, the discriminant validity of constructs showed the factor loading indicators on the assigned construct. Therefore, they had to be above all loading of other constructs (in the same column) with the condition that the cut-off value of factor loading was higher than 0.70 [89]. In addition, the model proved to have satisfactory reliability with convergent and discriminant validity. After this step, it was necessary to test the discriminant validity of constructs. According to Fornell and Larcker (1981) [89], with the correlation coefficient of the two dimensions less than the square root of the AVE, two dimensions were understood to have discriminant validity because of AVE > 0.5 (see Table 4).

The study confirmed the hypothesis with path coefficient, standard error, t-value, and p-value (see Table 5). It was concluded that all the hypotheses planted were supported and positive to predict the purchase intention with a high level, even though the study observed some differences about each association. The first force is the association between attitude toward the brand on purchase intention had the best path coefficient (*β* = 0.447). Moreover, the results showed that attitude toward eating a hamburger was also important to purchase intention (*β* = 0.197). However, the other association to predict purchase intention was throughout the positive anticipated emotions and for this model was (*β* = 0.206), more than attitude toward eating a hamburger. 

The great force to constitute the attitude toward the brand was with the construct positive anticipated emotions (*β* = 0.436). Because, in comparison, the attitude toward eating a hamburger only has *β* = 0.368. Something relevant was the impact of food values to the attitudes, where it had some consideration to attitude toward eating a hamburger (*β* = 0.270), in contrast to the brand, where was higher (*β* = 0.284). 

Some reflections about all the hypotheses proposed are the level of significance, where p-value <0.001 with the 99%; it means that these study results were statistically significant.

**Figure 2:**
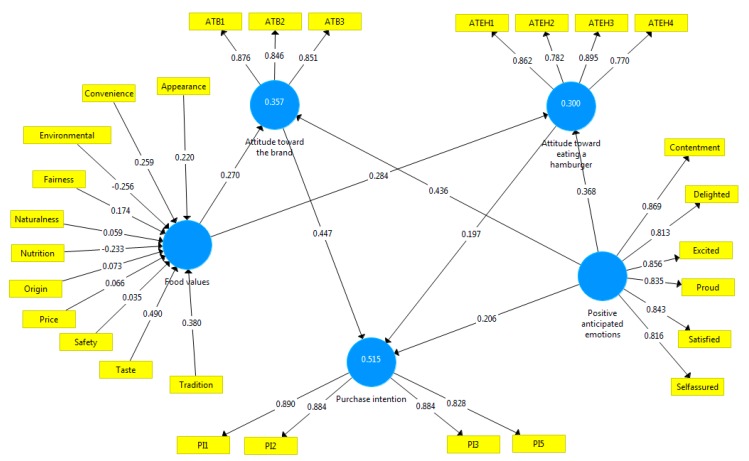
PLS analysis results.

Also, the H5 line of positive anticipated emotions to attitude toward the brand (*β* = 0.436, *t* = 10.126, *p* = < 0.001) and the H1 line of attitude to purchase intention (*β* = 0.447, *t* = 10.849, *p* = <0.001) indicated an abundant positive effect to form the purchase intention; this was the best way to predict it. Table 5 shows that in all the relations, t-value ≥ 1.96 and p-value ≤ 0.05; thus, this model supported all the hypotheses with high path coefficients and t-values. Hence, outer model loadings were highly significant. In addition, f^2^ was utilized to confirm the hypotheses null in the model and the outcomes supported each hypothesis but with different effects from weak <0.15 to large >0.15 [91]. All q2 are above zero, which supports the model presenting in Figure 2 [88].

Esposito et al. (2010) [92] stated that formative constructs need not be correlated between them. Also, the construct needs to be supported with the theory about food values. Similarly, the PLS algorithm produced loadings for reflective construct and weight for formative. Moreover, the study used the loadings and weights indicator for each construct by nature.

Figure 2 indicates the formative construct (food values), and inside the construct, the best items are taste and tradition (0.490; 0.380). On the other hand, the food values show negative loading with environment and nutrition (−0.256; −0.233). These facts do not have a position for the food value. Also, the model indicates that the emotions of contentment, excited and satisfied are the best loadings in the model (0.869, 0.856, 0.843).

It is distinguished that R^2^ (ATEH) is 0.357 higher than ATB (0.300). Additionally, R^2^ (PI) is 0.515, signifying that both attitudes toward eating and the brand plus positive anticipated emotions explain 51% of purchase intention. Even though R^2^-ATEH and R^2^-ATB are weak, the R^2^-purchase intention is substantial [91]. 

## 4. Discussion

All the hypotheses proposed were supported and confirmed. It accepted the difference by two types of attitudes: one of them toward the brand and the other toward eating a hamburger. Also, it showed the gap between the beta indicators with 0.250 to predict the purchase intention. The attitude toward the brand got first place in the hypotheses. Based on the previous study, the theory and empirical research suggested that attitude toward the brand will positively influence the intention to buy. After the results, it confirmed the positive influence and on the same road with other studies. In this case, it corroborated with the results of Hwang, Yoon and Park (2011) [29] which mentioned that the affective responses positively influence brand attitudes and purchase intention. The attitude toward eating had the right place in the final model. This hypothesis was confirmed, and the values obtained help to explain, with a higher percentage, the purchase intention. Other authors affirm the importance to investigate eating behavior to get knowledge about the positive or negative predisposition to eat [23,24]. The hypotheses related to food values were an essential variable in this model, i.e., the relationship of this variable to both attitudes. At this point, it is demonstrated that the food values could be impacted in a different way to each attitude. It validated the influence of food values affecting indirectly on the purchase intention. With this information, it led to some discussion to add more food values and to get an effect indirect to purchase intention. For example, these results match to Lang and Lemmerer (2019) [53] which affirm that personal values impact on forming a food attitude. Last, the positive anticipated emotion positively influenced attitude toward the brand, attitude toward eating, and intention to buy a hamburger. The results are consistent with previous research, which assert that emotion is an irreplaceable variable to try predicting the purchase intention. Positive anticipated emotion is a significant variable, which participates in three hypotheses addressing attitude toward the brand, attitude toward eating a hamburger and purchase intention. This confirms findings in other studies [74,93,94].

Managerial implications are confirmations derived from this research. First of all, managers of fast-food restaurants have to focus on the purchase intention of consumers. The findings support that purchase intention is more influenced by attitude toward the brand than by attitude toward eating a hamburger. Subsequently, the food values do not impact very strongly, but positive anticipated emotions do. The managers need to study how powerful each emotion (contentment, excited and satisfied) is before thinking about eating something at a fast-food restaurant. Also, the best values to build into the product are taste and tradition. Hence, in this case, the managers need to investigate about preferences, tastes and culture around consumption in fast-food restaurants. In that way, they need to prefer a strategy with a focus to increase and improve the value of the brand toward the brand equity oriented into the consumer. Correspondingly, positive anticipated emotions do not have a good association directly with purchase intention. This explains that without an attitude toward eating a hamburger or the attitude toward the brand, the consumer does not perceive the intention to buy a hamburger at a fast-food restaurant.

### Limitations and Future Orientations

There are limitations and suggested future lines of research. First of all, the sample should be increased to raise the level of confidence and lower the level of sampling error. Alternatively, it is recommended to add other variables related to TPB as perceived control, perceived difficulty and subjective norms on purchase intention. Finally, it is suggested to apply these surveys in other cities, products, and brands to know if there are significant differences between the samples. 

## 5. Conclusions

The goal for this study was building a development and testing model, having one comprehensive model about the purchase intention. The study planted a model with the importance of functional and emotional aspects through their effects on two attitudes. This model is an approximation to better explain the purchase intention. The food values have a low position on attitude toward the brand and attitude toward eating a hamburger. On the other hand, anticipated positive emotions have more relevance on attitudes, especially the attitude toward the brand and to purchase intention. 

The positive food values are taste and tradition in fast-food consumers. This model provides information to fast-food restaurants to pay attention to constantly evaluate the taste that has the consumers’ favor and to explore insights about a different perception of taste in the hamburger. Also, the tradition is significant because it includes and preserves traditional consumption patterns, since children families and reference groups help to educate this kind of consumption. From the other view, the consumer does not care about the nutrition of the hamburger against the knowledge of the brand. This confirms the results from Barone et al. (1996) [95] that examined the cause to form incorrect conclusions about the product. In this case, the consumer does not give value to the types of fat, proteins, vitamins, and carbohydrates that the hamburgers have. This demonstrates the lack of sensitivity and knowledge of healthy and responsible consumption.

Similarly, it is also happening with the environment value where the most significant weight in the variable of food value is. The consumer does not care if the burger is produced while taking care of the environment. The problem of having production for the environment and pollution does not see some or any benefit knowing how the food was manufactured. So, the adequacy of practices in favor of the environment and eco-friendly consumption is not significantly crucial for attitude or purchase intention. 

It was also shown that positive anticipated emotions form the best way to explain the purchase intention. First of all, it was verified that the anticipated negative emotions did not show any relevant data that included that variable within the model. Subsequently, the items with the greatest loadings were analyzed, and the results were positive anticipated emotions like contentment, delighted, excited, proud, satisfied, and self-assured. If the consumer is to have one of these emotions, it is probably to have a good level of attitude toward the brand and then to get a purchase intention. 

For this reason, the results of the study confirm the existence of a strong relationship between attitudes toward the brand on purchase intention by way of anticipated positive emotions in the consumer of fast-food restaurant. This proves, as in previous literature, that emotions are a necessary measure of the decision-making process of the consumer [96].

## Figures and Tables

**Table 1:** Technical Details.

Universe	Residents in Puebla State in México
Sample unit	People over 17 years old and buying fast food
Information collection method	Personal survey
Sample error	±4.335
Level of reliability	95%
Sample procedure	Probabilistic
Number surveyed	512 valid surveys
Period of information collection	January 26–May 23 (2018)
Language	Spanish

**Table 2:** Questionnaire sections.

Latent Variable	Observed Variables	Definition	Source
Food values are general food attributes that consumers believed were relatively more important when purchasing food	Appearance	Extent to which food looks appealing	Lusk (2011) [54]
	Convenience	Ease with which food is cooked and consumed	
	Environmental	Effect of food production on the environment	
	Fairness	The extent to which all parties involved in the production of the food equally benefit	
	Naturalness	Extent to which food is produced without modern technologies	
	Nutrition	Amount and type of fat, protein, vitamins, etc.	
	Origin	Where the agricultural commodities were grown	
	Price	The price that is paid for the food	
	Safety	Extent to which consumption of food will not cause illness	
	Taste	Extent to which consumption of the food is appealing to the senses	
	Tradition	Preserving traditional consumption patterns	
Positive and negative anticipated emotions	Contentment	If I can go to eat a hamburger in fast-food restaurants the next month, I feel contentment	Adapted from Bagozzi and Dholakia (2006) [79]
	Delighted	If I can go to eat a hamburger in fast-food restaurants the next month, I feel delighted	
	Excited	If I can go to eat a hamburger in fast-food restaurants the next month, I feel excited	
	Proud	If I can go to eat a hamburger in fast-food restaurants the next month, I feel proud	
	Satisfied	If I can go to eat a hamburger in fast-food restaurants the next month, I feel satisfied	
	Selfassured	If I can go to eat a hamburger in fast-food restaurants the next month, I feel self-assured	
Attitude toward the brand (ATB)	ATB1	Like the brand	Aggarwal and McGill (2012) [74]
	ATB2	Admire the brand	
	ATB3	Fit in your life the brand	
Attitude toward eating a hamburger (ATEH)	ATEH1	Eating the hamburger would be pleasurable	Adapted from Haws and Winterich (2013) [73]
	ATEH2	I would enjoy eating the hamburger	
	ATEH3	If I eat a hamburger, it would be satisfying for me	
	ATEH4	If I eat a hamburger because of the good taste it has	
Purchase intention	PI1	You probably buy products in fast-food restaurants	Adapted from Chiu, Hsieh, and Kuo (2012) [39], Diallo (2012) [40]
	PI2	I would consider buying a product in fast-food restaurants if I need a product of this type	
	PI3	It is possible to buy a product in fast-food restaurants	
	PI5	The probability that you consider buying in fast-food restaurants is high	

**Table 3:** Validity Testing.

	Cronbach’s Alpha Coefficient	rho_A	Composite Reliability (CR)	Average Variance Extracted (AVE)
Attitude toward eating a hamburger	0.847	0.862	0.897	0.687
Attitude toward the brand	0.822	0.836	0.893	0.736
Positive anticipated emotions	0.916	0.921	0.934	0.704
Purchase intention	0.895	0.896	0.927	0.760

**Table 4:** Association Testing.

	Attitude toward Eating a Hamburger	Attitude toward the Brand	Food Values	Positive Anticipated Emotions	Purchase Intention
Attitude toward eating a hamburger	0.829				
Attitude toward the brand	0.538	0.858			
Food values	0.431	0.444	Formative		
Positive anticipated emotions	0.482	0.544	0.401	0.839	
Purchase intention	0.537	0.665	0.407	0.544	0.872

**Table 5:** Hypothesis Testing and Path Coefficients.

		Beta	Standard Error	*t*-Value	*p*-Value	*f* ^2^	*q* ^2^	Supported
H1	Attitude toward the brand -> Purchase intention	0.447 ***	0.041	10.849	0.000	0.249	0.134	Yes
H2	Attitude toward eating a hamburger -> Purchase intention	0.197 ***	0.043	4.574	0.000	0.053	0.030	Yes
H3	Food values -> Attitude toward the brand	0.270 ***	0.042	6.447	0.000	0.095	0.050	Yes
H4	Food values -> Attitude toward eating a hamburger	0.284 ***	0.043	6.608	0.000	0.097	0.052	Yes
H5	Positive anticipated emotions -> Attitude toward the brand	0.436 ***	0.043	10.126	0.000	0.248	0.146	Yes
H6	Positive anticipated emotions -> Attitude toward eating a hamburger	0.368 ***	0.040	9.167	0.000	0.163	0.088	Yes
H7	Positive anticipated emotions -> Purchase intention	0.206 ***	0.050	4.129	0.000	0.057	0.030	Yes

Note: *n* = 10,000 subsamples; *** *p* < 0.001; R^2^ (Attitude toward the brand = 0.357; Attitude toward eating = 0.300; Purchase intention = 0.515); *q*^2^ = Predictive relevance calculated ((R-Sq included)-(Q-Sq excluded))/(1-R-Sq included).
